# Baseline peripheral blood immune markers associated with clinical outcomes in patients with advanced breast cancer treated with trastuzumab deruxtecan

**DOI:** 10.1186/s12885-026-16548-4

**Published:** 2026-07-13

**Authors:** Akihiro Fujimoto, Yuki Ichinose, Kazuo Matsuura, Emi Mikami, Wakako Inohana, Taeko Kurosawa, Ayako Nakame, Ayaka Sakakibara, Asami Nukui, Aya Asano, Hiroko Shimada, Hideki Yokogawa, Masahiro Ohara, Hiroshi Ishiguro, Takahiro Hasebe, Akihiko Osaki, Toshiaki Saeki

**Affiliations:** 1 Department of Breast Oncology, Saitama Medical University International Medical Center, 1397-1, Yamane, Hidaka, Saitama 350-1298 Japan; 2 Department of Breast Oncology, Saitama Medical University Hospital, 38 Moro-Hongo, Moroyama-machi, Iruma-gun, Saitama, 350-0495 Japan

**Keywords:** Breast cancer, Trastuzumab deruxtecan, Antibody–drug conjugate, Absolute lymphocyte count, Neutrophil-to-lymphocyte ratio

## Abstract

**Background:**

Trastuzumab deruxtecan (T-DXd) has demonstrated substantial clinical efficacy in patients with advanced breast cancer; however, predictive and prognostic biomarkers for treatment outcomes remain poorly defined. Host immune status has been reported to influence the efficacy of various systemic therapies, but its clinical significance in patients treated with T-DXd is not yet fully understood.

**Methods:**

We conducted a retrospective, single-center study of patients with breast cancer who received T-DXd. Their baseline peripheral blood immune markers—including absolute lymphocyte count (ALC), neutrophil-to-lymphocyte ratio (NLR), and platelet-to-lymphocyte ratio—were evaluated prior to treatment initiation. Time-to-treatment failure (TTF) and overall survival (OS) were analyzed using Kaplan–Meier methods and Cox proportional hazards models.

**Results:**

A total of 126 patients were included in the study. Univariate analyses showed that low ALC and high NLR were associated with shorter TTF and OS. In multivariate analyses adjusted for relevant clinical factors, low ALC and high NLR remained associated with shorter TTF and OS.

**Conclusions:**

Baseline peripheral blood immune markers, particularly ALC and NLR, may be associated with clinical outcomes in patients with breast cancer treated with T-DXd. These readily available markers may have potential clinical utility and warrant further prospective validation.

**Supplementary Information:**

The online version contains supplementary material available at https://doi.org/10.1186/s12885-026-16548-4.

## Background

Breast cancer is a biologically heterogeneous disease. Despite recent therapeutic advances in its management, treatment resistance and disease progression remain major clinical challenges in advanced settings [1, 2]. The development of antibody-drug conjugates (ADCs) has substantially changed the treatment landscape for this malignancy, facilitating the selective delivery of cytotoxic agents to tumor cells while limiting systemic toxicity [3].

Trastuzumab deruxtecan (T-DXd) is a novel ADC composed of an anti-human epidermal growth factor receptor 2 (HER2) monoclonal antibody linked to a topoisomerase I inhibitor payload [4]. It has demonstrated significant clinical efficacy in patients with HER2-positive advanced breast cancer (ABC) and in those with HER2-low disease, leading to its widespread use across multiple treatment lines [5–8]. However, substantial interpatient variability in treatment responses and durability has been observed in real-world clinical practice, and reliable predictive or prognostic biomarkers for T-DXd have yet to be established [9].

Host immune status has emerged as an important determinant of treatment outcomes in various systemic therapies for advanced cancer. Peripheral blood immune markers such as absolute lymphocyte count (ALC), neutrophil-to-lymphocyte ratio (NLR), and platelet-to-lymphocyte ratio (PLR) have been reported to be associated with treatment response or survival outcomes in patients with ABC who receive cytotoxic chemotherapy, including eribulin [10–14]. Furthermore, similar associations have also been reported in patients who receive molecular-targeted therapies such as cyclin-dependent kinase 4 and 6 (CDK4/6) inhibitors or bevacizumab [15–17], indicating that host immune competence influences treatment outcomes even in non-immunotherapeutic settings.

However, the clinical significance of baseline peripheral blood immune markers in patients treated with T-DXd remains unclear. Therefore, we aimed to investigate the association between baseline peripheral immune markers, including ALC, NLR, and PLR, and clinical outcomes in patients with ABC who were treated using T-DXd. We specifically evaluated their impact on time-to-treatment failure (TTF) and overall survival (OS), to clarify the potential clinical relevance of host immune status as a practical and readily available biomarker in the ADC era.

## Methods

### Patients

In this retrospective study, we enrolled patients with ABC who received T-DXd at Saitama Medical University International Medical Center between July 2020 and December 2025. Patients were eligible if they had histologically confirmed breast cancer and received T-DXd as any line of systemic therapy for metastatic disease. Metastatic disease was confirmed by diagnostic imaging, which included computed tomography, magnetic resonance imaging, bone scintigraphy, or positron emission tomography, as appropriate. Patients with concurrent active malignancies other than breast cancer were excluded. Clinicopathological characteristics and clinical outcome data were retrospectively extracted from the center’s electronic medical records.

### Treatment procedures

T-DXd was administered intravenously at a standard dose of 5.4 mg/kg every 3 weeks, in accordance with the approved regimen. Treatment was continued until disease progression, unacceptable toxicity, or death. In cases of treatment-related adverse events, dose interruption, dose reduction, or treatment delay was performed at the discretion of the treating physician, according to institutional guidelines and the approved dose modification protocol.

### Clinicopathological assessment

All of the patients included in this study were histologically diagnosed with breast cancer. The clinicopathological variables evaluated at baseline included age at T-DXd initiation, Eastern Cooperative Oncology Group performance status (ECOG PS), disease status at metastatic diagnosis (*de novo* metastatic or recurrent disease), estrogen receptor (ER) status, HER2 expression status (HER2-positive or HER2-low), number of prior treatment regimens for metastatic or recurrent disease, history of perioperative chemotherapy, prior exposure to trastuzumab and pertuzumab, prior exposure to trastuzumab emtansine (T-DM1), prior exposure to CDK4/6 inhibitors, endocrine resistance status, and metastatic sites at T-DXd initiation. The number of prior treatment regimens for metastatic disease was determined based on previously administered systemic therapies. Endocrine therapy, CDK4/6 inhibitor-containing regimens, and immune checkpoint inhibitor-containing regimens were included in the count. Endocrine resistance was defined as disease progression after endocrine therapy-containing regimens administered for metastatic or recurrent disease.

ER and progesterone receptor positivity were defined as ≥ 1% nuclear staining of tumor cells by immunohistochemistry (IHC), in accordance with the American Society of Clinical Oncology/College of American Pathologists (ASCO/CAP) guidelines [18]. HER2 positivity was defined as an IHC score of ≥ 3 or an IHC score of ≥ 2 with HER2 gene amplification confirmed by in situ hybridization (ISH). HER2-low expression was defined as an IHC score of ≥ 2 with negative HER2 amplification as assessed by ISH, or an IHC score of ≥ 1. HER2 status was assessed according to the ASCO/CAP guidelines [19]. IHC was performed to assess HER2 status, using clinically approved companion diagnostic antibodies—including anti-HER2 antibody clone 4B5 (Ventana Medical Systems; Roche Diagnostics)—as appropriate.

### Peripheral blood immune markers

Baseline peripheral blood samples were obtained on the day of T-DXd administration, prior to initiation of the first treatment cycle. Absolute neutrophil counts, lymphocyte counts, and platelet counts were measured using an automated hematology analyzer at the study center. ALC was evaluated as an independent parameter. NLR was calculated by dividing the absolute neutrophil count by the ALC, and PLR was calculated by dividing the platelet count by the ALC. Based on protocols used in previous related studies, the patients were stratified into high and low groups using predefined cutoff values for baseline immune markers [10–12, 14, 20]. ALC was categorized as low (< 1,500/µL) or high (≥ 1,500/µL); NLR was categorized as low (< 3.0) or high (≥ 3.0); and PLR was also categorized as low (< 150) or high (≥ 150). These cutoff values were determined a priori and were not optimized based on the present dataset, to minimize potential overfitting and selection bias [14].

### Clinical outcomes

The clinical outcomes evaluated in this study were TTF and OS. TTF was defined as the interval from the initiation of T-DXd until treatment discontinuation for any reason, including disease progression, adverse events, or death. OS was defined as the interval from T-DXd initiation until death from any cause or the final follow-up date.

### Statistical analysis

Continuous variables are presented as medians with associated ranges, whereas categorical variables are presented as numbers and percentages. Survival curves for TTF and OS were estimated using the Kaplan–Meier method, and differences between groups were compared using the log-rank test. Associations between clinicopathological variables, peripheral blood immune markers, and clinical outcomes were evaluated using Cox proportional hazards regression models. The proportional hazards assumption was evaluated using the Schoenfeld residual test, and no significant violations were observed. Clinically relevant covariates prespecified based on prior literature and clinical relevance, were included in the multivariable analyses regardless of univariable significance. These covariates included age, ECOG PS, HER2 status (HER2-positive vs. HER2-low), ER status (positive vs. negative), number of prior treatment regimens for metastatic or recurrent disease, prior exposure to anti-HER2 therapy (including trastuzumab/pertuzumab, and T-DM1), and prior CDK4/6 inhibitor exposure. In addition, variables with *P* < 0.10 in univariable analyses were also considered for multivariable analyses. In the multivariable analyses, ECOG PS was categorized as 0–1 vs. others, with patients with missing ECOG PS data included in the latter category. No patients were excluded owing to missing data for baseline immune markers, other covariates, or survival outcomes. Because ALC is mathematically incorporated into NLR and PLR, these variables were not simultaneously included in the same multivariable model to avoid redundancy and potential multicollinearity. Prior perioperative chemotherapy was excluded because of its close association with recurrent vs. *de novo* metastatic presentation. Endocrine resistance status was also excluded from the multivariable models because this variable was applicable only to patients with ER-positive disease. Exploratory interaction analyses were performed using Cox proportional hazards models, including breast cancer subtype, peripheral blood immune markers (ALC and NLR), and interaction terms between each marker and subtype, with adjustment for the same covariates as in the primary multivariable analyses. Any two-sided *P* values of < 0.05 were considered statistically significant. Statistical analyses were performed using EZR version 1.52 (Saitama Medical Center, Jichi Medical University, Saitama, Japan), a graphical user interface for R software (The R Foundation for Statistical Computing, Vienna, Austria).

## Results

### Patient characteristics

The baseline clinicopathological characteristics of the 126 patients treated with T-DXd are summarized in Table 1. The median age was 61 years (range, 28–91 years). ECOG PS was 0–1 in 101 patients (80.2%) and 2–3 in 12 patients (9.5%). Fifty patients (39.7%) had *de novo* metastatic disease, whereas 76 (60.3%) had recurrent disease. ER-positive disease was observed in 92 patients (73.0%), and 67 patients (53.2%) had HER2-positive disease, whereas 59 (46.8%) had HER2-low disease. T-DXd was administered after 0–2 prior treatment regimens for metastatic disease in 63 patients (50.0%) and after ≥ 3 prior regimens in the remaining 63 patients (50.0%). Prior exposure to trastuzumab and pertuzumab, T-DM1, and CDK4/6 inhibitors was documented in 61 (48.4%), 40 (31.7%), and 43 patients (34.1%), respectively. Regarding metastatic sites, liver metastases were present in 59 patients (46.8%), lung or pleural metastases in 65 (51.6%), and brain metastases in 20 (15.9%). Baseline peripheral blood immune marker assessment showed low ALC (< 1,500/µL) in 86 (68.3%), high NLR (≥ 3.0) in 50 (44.4%), and high PLR (≥ 150) in 79 patients (62.7%).

**Table 1:** Patient characteristics (*n* = 126)

Variables	*n* (%)
Median age, years (range)	61 (28–91)
Age, years	
< 50	26 (20.6%)
≥ 50	100 (79.4%)
ECOG PS	
0–1	101 (80.2%)
2–3	12 (9.5%)
unknown	13 (10.3%)
Diagnosis	
* De novo*	50 (39.7%)
Recurrence	76 (60.3%)
ER status	
Negative	34 (27.0%)
Positive	92 (73.0%)
HER2 status	
HER2-low	59 (46.8%)
HER2-positive	67 (53.2%)
Number of prior treatment regimens for metastatic disease	
0–2	63 (50.0%)
≥ 3	63 (50.0%)
Prior perioperative chemotherapy	
No	65 (51.6%)
Yes	61 (48.4%)
Prior trastuzumab/pertuzumab exposure	
No	65 (51.6%)
Yes	61 (48.4%)
Prior T-DM1 exposure	
No	86 (68.3%)
Yes	40 (31.7%)
Prior CDK4/6 inhibitor exposure	
No	83 (65.9%)
Yes	43 (34.1%)
Endocrine resistance status	
No	73 (57.9%)
Yes	53 (42.1%)
Liver metastases	
Negative	67 (53.2%)
Positive	59 (46.8%)
Lung or pleural metastases	
Negative	61 (48.4%)
Positive	65 (51.6%)
Brain metastases	
Negative	106 (84.1%)
Positive	20 (15.9%)
Absolute lymphocyte count (ALC)	
Low (< 1500)	86 (68.3%)
High (≥ 1500)	40 (31.7%)
Neutrophil-to-lymphocyte ratio (NLR)	
Low (< 3)	70 (55.6%)
High (≥ 3)	56 (44.4%)
Platelet-to-lymphocyte ratio (PLR)	
Low (< 150)	47 (37.3%)
High (≥ 150)	79 (62.7%)

*Abbreviations ALC* Absolute lymphocyte count, *CDK4/6* Cyclin-dependent kinase 4 and 6, *ECOG PS* Eastern Cooperative Oncology Group performance status, *ER* Estrogen receptor, *HER2* human epidermal growth factor receptor 2, *NLR* Neutrophil-to-lymphocyte ratio, *PLR* Platelet-to-lymphocyte ratio, *T-DM1* trastuzumab emtansine

### Treatment outcomes

During the follow-up period, TTF events occurred in 98 patients and death events occurred in 39 patients. The median follow-up duration was 23.2 months. The median TTF and OS in the overall cohort were 10.8 months (95% confidence interval [CI], 8.0–14.0) and 38.6 months (95% CI, 31.5–45.8), respectively. The reasons for T-DXd discontinuation are summarized in Additional file 1, Supplementary Table S1. Disease progression was the most common reason for treatment discontinuation, accounting for 72.4% of all discontinuations, followed by adverse events (17.3%), other reasons (9.2%), and death (1.0%). Similar patterns were observed across the ALC and NLR subgroups, with disease progression remaining the predominant cause of treatment discontinuation in all groups.

### Association between baseline peripheral blood immune markers and TTF

**Fig. 1:**
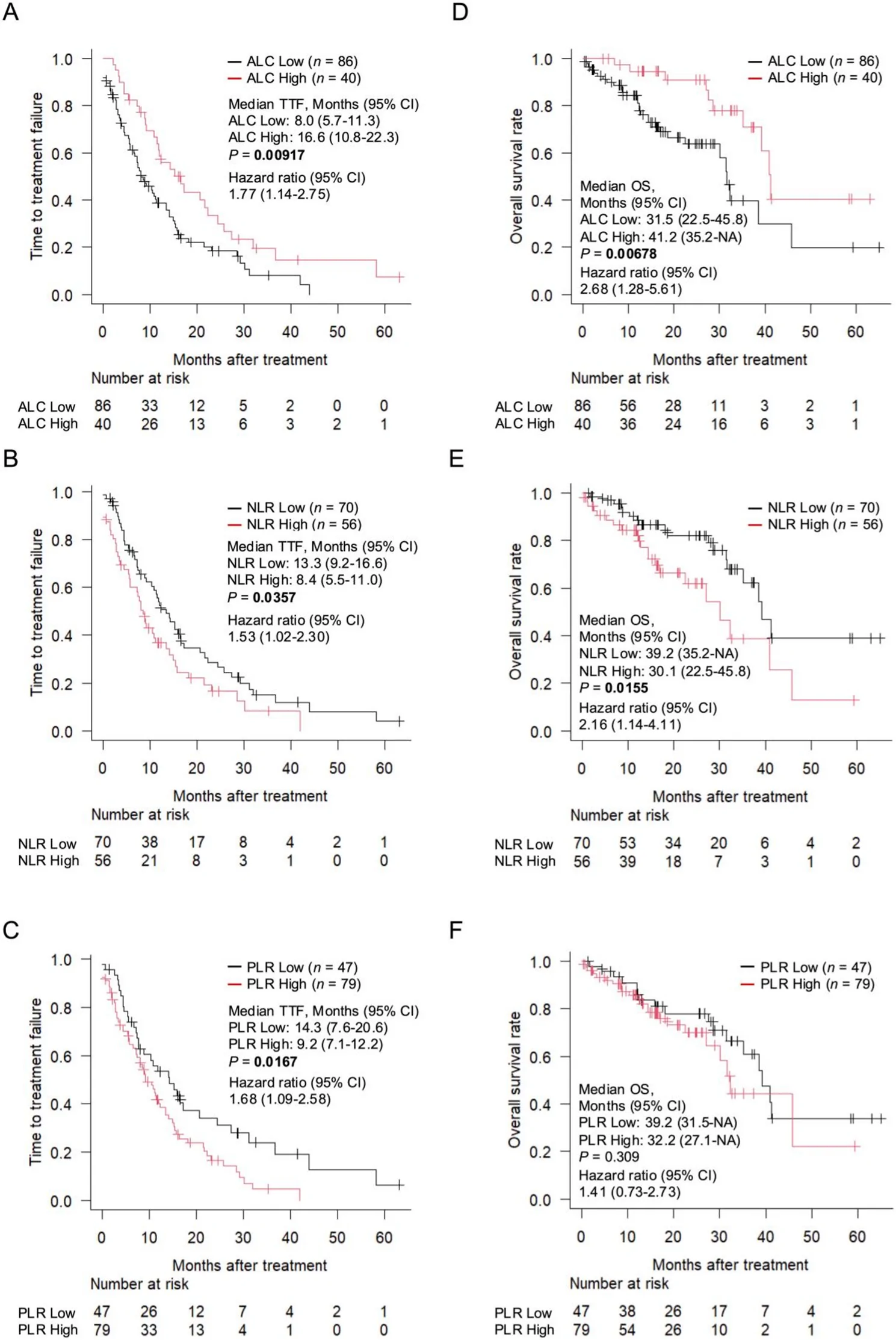
Association between baseline peripheral blood immune markers and clinical outcomes in patients treated with T-DXd. **A–C** Kaplan–Meier curves of time-to-treatment failure (TTF) according to baseline absolute lymphocyte count (ALC; high vs. low), neutrophil-to-lymphocyte ratio (NLR; high vs. low), and platelet-to-lymphocyte ratio (PLR; high vs. low). High and low groups were defined using the pre-specified cutoff values, resulting in the following numbers of patients in each group: ALC high, *n* = 40; ALC low, *n* = 86; NLR high, *n* = 56; NLR low, *n* = 70; PLR high, *n* = 79; PLR low, *n* = 47. **D–F** Kaplan–Meier curves of overall survival (OS) stratified by the same ALC, NLR, and PLR categories used in panels **A–C**. *P* values were calculated using the log-rank test. CI, confidence interval; T-DXd, trastuzumab deruxtecan

Kaplan–Meier analysis demonstrated that the patients with low ALC experienced significantly shorter TTF than those with high ALC (*P* = 0.00917) (Fig. 1A). Similarly, the patients with high NLR showed significantly shorter TTF than those with low NLR (*P* = 0.0357) (Fig. 1B). High PLR was also associated with shorter TTF (*P* = 0.0167) (Fig. 1C). Univariate Cox proportional hazards analysis revealed that low ALC, high NLR, and high PLR were all associated with shorter TTF (Table 2). ECOG PS, HER2 status, prior trastuzumab/pertuzumab exposure, prior T-DM1 exposure, prior CDK4/6 inhibitor exposure, and endocrine resistance status were also associated with TTF in univariate analysis (Table 2). In multivariate Cox proportional hazards analysis, low ALC was associated with shorter TTF (Table 2). To further evaluate potential multicollinearity between immune markers, an additional multivariate model incorporating NLR instead of ALC was constructed. In this analysis, consistent with the findings for ALC, NLR remained associated with TTF (Additional file 1, Supplementary Table S2). Subgroup analyses according to breast cancer subtype—including HER2-positive disease, HER2-low ER-positive disease, and HER2-low triple-negative disease—are shown in Additional file 1, Supplementary Figures S1–S3. No statistically significant associations between baseline peripheral blood immune markers and TTF were observed within any of the individual subtype subgroups. Consistently, interaction analyses did not demonstrate statistically significant heterogeneity according to subtype (ALC: *P* for interaction = 0.6317; NLR: *P* for interaction = 0.3077).

**Table 2:** Univariate and multivariate analyses of clinicopathological parameters and time-to-treatment failure

Variables	Univariate			Multivariate		
	HR	95% CI	*P*-value	HR	95% CI	*P*-value
Time-to-treatment failure						
Age (< 50 vs. ≥50 years)	0.99	0.60–1.65	0.9821	0.97	0.59–1.67	0.9697
ECOG PS (0–1 vs. others)	0.59	0.35–0.98	**0.0432**	0.70	0.41–1.20	0.1966
Diagnosis (Recurrence vs. *De novo*)	0.83	0.56–1.24	0.3673			
ER status (+/-)	1.31	0.82–2.11	0.2566	1.12	0.67–1.88	0.6695
HER2 status (HER2-low vs. HER2-positive)	2.27	1.48–3.48	**0.0002**	1.21	0.45–3.27	0.7055
Number of prior treatment regimens for metastatic disease (≥ 3 vs. 0–2)	1.09	0.73–1.63	0.6836	1.19	0.70–2.03	0.5153
Prior perioperative chemotherapy (Yes vs. No)	0.96	0.64–1.43	0.8301			
Prior trastuzumab/pertuzumab exposure (Yes vs. No)	0.41	0.27–0.64	**< 0.0001**	0.43	0.16–1.17	*0.0975*
Prior T-DM1 exposure (Yes vs. No)	0.63	0.40–0.98	**0.0421**	0.69	0.35–1.35	0.2729
Prior CDK4/6 inhibitor exposure (Yes vs. No)	1.70	1.10–2.64	**0.0177**	1.00	0.49–2.04	0.9971
Endocrine resistance status (Yes vs. No)	1.67	1.10–2.55	**0.0162**			
Liver metastases (+/-)	1.07	0.72–1.59	0.7546			
Lung or pleural metastases (+/-)	1.02	0.68–1.52	0.9206			
Brain metastases (+/-)	1.01	0.59–1.73	0.9752			
ALC (Low vs. High)	1.77	1.14–2.75	**0.0105**	1.61	1.02–2.54	**0.0422**
NLR (High vs. Low)	1.53	1.02–2.30	**0.0381**			
PLR (High vs. Low)	1.68	1.09–2.58	**0.0185**			

*Abbreviations ALC* Absolute lymphocyte count, *CDK4/6* Cyclin-dependent kinase 4 and 6, *CI* Confidence interval, *ECOG PS* Eastern Cooperative Oncology Group performance status, *ER* Estrogen receptor, *HER2* Human epidermal growth factor receptor 2, *HR* Hazard ratio, *NLR* Neutrophil-to-lymphocyte ratio, *OS* Overall survival, *PLR* Platelet-to-lymphocyte ratio, *T-DM1* trastuzumab emtansine

### Association between baseline peripheral blood immune markers and OS

The OS analysis yielded findings that were generally consistent with those observed for TTF. Patients with low ALC had significantly shorter OS than those with high ALC (*P* = 0.00678) (Fig. 1D). Patients with high NLR also had significantly shorter OS than those with low NLR (*P* = 0.0155) (Fig. 1E), whereas PLR was not significantly associated with OS (Fig. 1F). In univariate Cox proportional hazards analysis, low ALC, high NLR, the presence of liver metastasis, and the presence of prior trastuzumab/pertuzumab exposure were associated with shorter OS (Table 3). In multivariate analysis, low ALC remained associated with shorter OS (Table 3). Similar to the TTF analysis, an additional multivariate model incorporating NLR instead of ALC was also constructed. In this analysis, consistent with the findings for ALC, NLR remained associated with OS (Additional file 1, Supplementary Table S3). Subgroup analyses according to breast cancer subtype—including HER2-positive disease, HER2-low ER-positive disease, and HER2-low triple-negative disease—are shown in Additional file 1, Supplementary Figures S1–S3. In the HER2-positive subgroup, low ALC and high NLR were associated with shorter OS, whereas no statistically significant associations between baseline peripheral blood immune markers and OS were observed in the HER2-low ER-positive or HER2-low triple-negative subgroups. However, interaction analyses did not demonstrate statistically significant heterogeneity according to subtype (ALC: *P* for interaction = 0.8085; NLR: *P* for interaction = 0.3527).

**Table 3:** Univariate and multivariate analyses of clinicopathological parameters and overall survival

Variables	Univariate			Multivariate		
	HR	95% CI	*P*-value	HR	95% CI	*P*-value
Overall survival						
Age (< 50 vs. ≥50 years)	1.88	0.96–3.66	*0.0649*	1.52	0.70–3.30	0.2877
ECOG PS (0–1 vs. others)	0.51	0.21–1.25	0.1412	0.59	0.24–1.48	0.2629
Diagnosis (Recurrence vs. *De novo*)	0.75	0.40–1.42	0.3765			
ER status (+/-)	1.05	0.53–2.10	0.8861	1.33	0.62–2.87	0.4635
HER2 status (HER2-low vs. HER2-positive)	2.08	0.97–4.42	*0.0582*	1.00	0.17–5.97	0.9973
Number of prior treatment regimens for metastatic disease (≥ 3 vs. 0–2)	1.05	0.55–1.98	0.8922	0.97	0.43–2.19	0.9491
Prior perioperative chemotherapy (Yes vs. No)	0.78	0.41–1.49	0.4463			
Prior trastuzumab/pertuzumab exposure (Yes vs. No)	0.46	0.22–0.97	**0.0407**	0.20	0.04–1.04	*0.0561*
Prior T-DM1 exposure (Yes vs. No)	0.88	0.42–1.85	0.7289	0.38	0.09–1.51	0.1680
Prior CDK4/6 inhibitor exposure (Yes vs. No)	1.36	0.63–2.96	0.4345	1.48	0.47–4.73	0.5040
Endocrine resistance status (Yes vs. No)	1.29	0.65–2.55	0.4703			
Liver metastases (+/-)	2.34	1.21–4.50	**0.0112**	1.81	0.85–3.89	0.1263
Lung or pleural metastases (+/-)	1.11	0.59–2.12	0.7405			
Brain metastases (+/-)	1.57	0.74–3.32	0.2396			
ALC (Low vs. High)	2.68	1.28–5.61	**0.0089**	2.32	1.04–5.18	**0.0394**
NLR (High vs. Low)	2.16	1.14–4.11	**0.0180**			
PLR (High vs. Low)	1.41	0.73–2.73	0.3108			

*Abbreviations ALC* Absolute lymphocyte count, *CDK4/6* Cyclin-dependent kinase 4 and 6, *CI* confidence interval, *ECOG PS* Eastern Cooperative Oncology Group performance status, *ER* estrogen receptor, *HER2* human epidermal growth factor receptor 2, *HR* hazard ratio, *NLR* neutrophil-to-lymphocyte ratio, *OS* overall survival, *PLR* platelet-to-lymphocyte ratio, *T-DM1* trastuzumab emtansine

### Sensitivity analyses of peripheral blood immune markers using continuous variables

To assess the robustness of the findings obtained using predefined cutoff values, sensitivity analyses were performed by modeling peripheral blood immune markers as a continuous variable. The results were generally consistent with those of the primary analyses (Additional file 1, Supplementary Table S4).

## Discussion

To our knowledge, this retrospective study represents one of the largest analyses to date focusing on the association between baseline peripheral blood immune markers and clinical outcomes in patients with breast cancer treated with T-DXd. In the present study, low ALC and high NLR were associated with shorter TTF and OS in univariate and multivariate analyses. These findings suggest that baseline host immune status is associated with treatment durability and survival outcomes in patients receiving T-DXd. The identification of reliable prognostic or predictive biomarkers for ADCs remains an unmet clinical need [21]. Our results suggest that readily available peripheral blood immune markers help in risk stratification for patients treated with T-DXd.

ALC, NLR, and PLR, which indicate systemic immune status, have been reported to represent significant prognostic factors for breast cancer [22]. Previous studies have reported that higher ALC and lower NLR are associated with improved survival in patients who are treated with cytotoxic chemotherapy, including eribulin [10, 11, 13]. Notably, a *post hoc* analysis of data from the EMBRACE trial demonstrated that high baseline ALC was associated with longer OS in patients who were treated with eribulin, whereas this association was not observed in patients who received treatments selected by their attending physician, suggesting a treatment-specific interaction between host immunity and therapeutic efficacy [12]. Notably, high baseline NLR was associated with poorer survival outcomes in the eribulin and physician’s choice groups in the EMBRACE trial, indicating that NLR may function as a general prognostic marker rather than a treatment-specific factor. Our findings are consistent with the observations in patients treated with T-DXd and further support the notion that systemic immune status may be associated with clinical outcomes across different systemic therapies.

In contrast to ALC and NLR, the prognostic value of PLR was less consistent in the present study. PLR was associated with TTF but not with OS, suggesting that it primarily reflects systemic inflammatory or platelet-related processes that influence early treatment discontinuation rather than long-term survival [23–25]. Collectively, these findings suggest that lymphocyte-related immune markers are associated with clinical outcomes in patients treated with T-DXd.

Although T-DXd is not traditionally classified as an immunotherapy, accumulating evidence suggests that ADCs exert immunomodulatory effects [26, 27]. The topoisomerase I inhibitor payload of T-DXd has been shown to induce immunogenic cell death, resulting in the release of tumor-associated antigens and damage-associated molecular patterns that can promote antitumor immune responses [28–30]. In addition, the bystander effect characteristic of T-DXd may further enhance tumor cell killing and facilitate antigen presentation within the tumor microenvironment [29–32]. In this context, an adequate pool of functional lymphocytes may be required to translate these immunogenic signals into effective antitumor immunity. Conversely, baseline lymphopenia may limit the immune-mediated amplification of ADC efficacy, potentially leading to reduced clinical outcomes.

Recent studies have also reported the relationship between immune status and outcomes in patients treated with ADCs [20, 33]. Togashi et al. [20] reported that ALC assessed at the end of treatment was associated with survival outcomes in patients with HER2-low disease. In this study, conversely, we focused on pre-treatment immune status, demonstrating the association of baseline ALC and NLR for TTF and OS in a larger patient cohort that was treated exclusively with T-DXd. These differences may reflect variations in study design, patient population, and endpoints—but collectively suggest that immune status at multiple time points during treatment may be clinically relevant. Baseline markers have the notable advantage of being available prior to treatment initiation, and may therefore be more useful for upfront clinical decision-making.

In exploratory subgroup analyses, low ALC and high NLR were associated with shorter OS in the HER2-positive subgroup, whereas no statistically significant associations were observed in the HER2-low ER-positive or HER2-low triple-negative subgroups. These subgroup analyses were limited by the relatively small sample sizes, particularly in the HER2-low triple-negative subgroup, and should therefore be interpreted cautiously. To further assess potential heterogeneity across subtypes, interaction analyses incorporating subtype and interaction terms between peripheral blood immune markers and subtype were performed; however, no statistically significant interactions were observed. Further validation in larger cohorts is warranted.

In the present study, TTF was selected as a clinically relevant endpoint because it captures treatment discontinuation in routine practice, including discontinuation owing to disease progression, adverse events, and death. However, because TTF incorporates multiple causes of treatment cessation, its biological interpretation requires caution. In the present cohort, disease progression accounted for approximately 70% of treatment discontinuations and was the predominant reason for discontinuation across all ALC and NLR subgroups. These findings suggest that the associations between low ALC, high NLR, and shorter TTF largely reflect inferior disease control, although the potential contribution of treatment-related toxicity cannot be fully excluded.

Notably, because this study lacked a comparator cohort treated with alternative systemic therapies, we cannot determine whether baseline ALC and NLR represent biomarkers specifically predictive of T-DXd efficacy or more general prognostic indicators reflecting host immune competence and tumor burden. Given that similar associations have been reported across multiple cancer types and treatment modalities, including chemotherapy, HER2-targeted therapy, and other antibody–drug conjugates, our findings are more consistent with a general prognostic role of systemic immune status rather than with a treatment-specific predictive effect for T-DXd.

Some limitations of this study should be acknowledged. First, its retrospective, single-center design likely introduced the possibility of selection bias and unmeasured confounding. Second, although multivariable analyses were adjusted for clinically relevant covariates, residual confounding from unmeasured or incompletely measured factors cannot be excluded owing to the observational nature of the study. Several potentially important variables, including disease burden, steroid use, concurrent infection or inflammatory conditions, dose intensity, and dose modification patterns, were not available. Therefore, baseline ALC and NLR may reflect treatment-related immune status and general patient condition, disease severity, nutritional status, bone marrow reserve, or systemic inflammation. Third, the number of OS events was relatively limited, which may have reduced statistical power and affected the stability of the multivariable estimates. Fourth, immune markers were assessed only at the baseline, and dynamic changes in them were not evaluated during the treatment period. In addition, tumor-infiltrating immune cells and other components of the tumor immune microenvironment were not assessed. Finally, information regarding subsequent treatments after T-DXd was limited, which may have influenced the OS outcomes.

## Conclusions

Our findings suggest that baseline peripheral blood immune markers are associated with clinical outcomes, highlighting the importance of host immunity in the ADC era. Assessing peripheral immune status prior to the initiation of T-DXd treatment may contribute to risk stratification and hypothesis generation for future biomarker-driven studies. Prospective studies are warranted to validate these findings and further elucidate the interaction between ADC therapy and host immunity.

## Supplementary Information

Supplementary Material 1.

## Data Availability

The datasets used and/or analyzed during the current study are available from the corresponding author upon reasonable request.

## Abbreviations

T-DXd: Trastuzumab deruxtecan
ALC: Absolute lymphocyte count
NLR: Neutrophil-to-lymphocyte ratio
TTF: Time-to-treatment failure
OS: Overall survival
ADCs: Antibody-drug conjugates
HER2: Human epidermal growth factor receptor 2
ABC: Advanced breast cancer
PLR: Platelet-to-lymphocyte ratio
CDK4/6: Cyclin-dependent kinase 4 and 6
ECOG PS: Eastern cooperative oncology group performance status
ER: Estrogen receptor
T-DM1: Trastuzumab emtansine
IHC: Immunohistochemistry
ASCO/CAP: American society of clinical oncology/college of american pathologists
ISH: In situ hybridization

## References

[1] Harbeck N, Penault-Llorca F, Cortes J, Gnant M, Houssami N, Poortmans P, et al. Breast cancer. Nat Rev Dis Primers. 2019;5:66. 10.1038/s41572-019-0111-2 31548545

[2] Loibl S, Poortmans P, Morrow M, Denkert C, Curigliano G. Breast cancer. Lancet. 2021;397:1750–69. 10.1016/S0140-6736(20)32381-3 33812473

[3] Tarantino P, Ricciuti B, Pradhan SM, Tolaney SM. Optimizing the safety of antibody-drug conjugates for patients with solid tumours. Nat Rev Clin Oncol. 2023;20:558–76. 10.1038/s41571-023-00783-w 37296177

[4] Martín M, Pandiella A, Vargas-Castrillón E, Díaz-Rodríguez E, Iglesias-Hernangómez T, Martínez Cano C, et al. Trastuzumab deruxtecan in breast cancer. Crit Rev Oncol Hematol. 2024;198:104355. 10.1016/j.critrevonc.2024.104355 38621469

[5] Modi S, Saura C, Yamashita T, Park YH, Kim SB, Tamura K, et al. Trastuzumab deruxtecan in previously treated HER2-positive breast cancer. N Engl J Med. 2020;382:610–21. 10.1056/NEJMoa1914510 PMC7458671 31825192

[6] Modi S, Jacot W, Yamashita T, Sohn J, Vidal M, Tokunaga E, et al. Trastuzumab deruxtecan in previously treated HER2-low advanced breast cancer. N Engl J Med. 2022;387:9–20. 10.1056/NEJMoa2203690 PMC10561652 35665782

[7] André F, Hee Park Y, Kim SB, Takano T, Im SA, Borges G, et al. Trastuzumab deruxtecan versus treatment of physician’s choice in patients with HER2-positive metastatic breast cancer (DESTINY-Breast02): A randomised, open-label, multicentre, phase 3 trial. Lancet. 2023;401:1773–85. 10.1016/S0140-6736(23)00725-0 37086745

[8] Yang C, Brezden-Masley C, Joy AA, Sehdev S, Modi S, Simmons C, et al. Targeting HER2-low in metastatic breast cancer: an evolving treatment paradigm. Ther Adv Med Oncol. 2023;15:17588359231175440. 10.1177/17588359231175440 PMC10262633 37323186

[9] Xue C, Liao Q, Huang R, Huang Y, Chen R, Yang Z, et al. Prognostic index for predicting outcomes of trastuzumab deruxtecan in HER2 expressing metastatic breast cancer: a real-world multicenter study. Oncologist. 2025;30:oyaf174. 10.1093/oncolo/oyaf174 PMC12345626 40589051

[10] Araki K, Ito Y, Fukada I, Kobayashi K, Miyagawa Y, Imamura M, et al. Predictive impact of absolute lymphocyte counts for progression-free survival in human epidermal growth factor receptor 2-positive advanced breast cancer treated with pertuzumab and trastuzumab plus eribulin or nab-paclitaxel. BMC Cancer. 2018;18:982. 10.1186/s12885-018-4888-2 PMC6191898 30326862

[11] Miyagawa Y, Araki K, Bun A, Ozawa H, Fujimoto Y, Higuchi T, et al. Significant association between low baseline neutrophil-to-lymphocyte ratio and improved progression-free survival of patients with locally advanced or metastatic breast cancer treated with eribulin but not with nab-paclitaxel. Clin Breast Cancer. 2018;18:400–9. 10.1016/j.clbc.2018.03.002 29605174

[12] Miyoshi Y, Yoshimura Y, Saito K, Muramoto K, Sugawara M, Alexis K, et al. High absolute lymphocyte counts are associated with longer overall survival in patients with metastatic breast cancer treated with eribulin-but not with treatment of physician’s choice-in the EMBRACE study. Breast Cancer. 2020;27:706–15. 10.1007/s12282-020-01067-2 PMC7297864 32133606

[13] Nakamoto S, Ikeda M, Kubo S, Yamamoto M, Yamashita T. Dynamic changes in absolute lymphocyte Counts During eribulin therapy are associated with survival benefit. Anticancer Res. 2021;41:3109–19. 10.21873/anticanres.15095 34083304

[14] Shimada H, Fujimoto A, Matsuura K, Kohyama S, Nukui A, Ichinose Y, et al. Comprehensive prognostic prediction of metastatic breast cancer treated with eribulin using blood-based parameters and ratio. Mol Clin Oncol. 2024;20:15. 10.3892/mco.2024.2713 PMC10809355 38274088

[15] Nakamoto S, Ikeda M, Kubo S, Yamamoto M, Yamashita T, Notsu A. Systemic immunity markers associated with lymphocytes predict the survival benefit from paclitaxel plus bevacizumab in HER2 negative advanced breast cancer. Sci Rep. 2021;11:6328. 10.1038/s41598-021-85948-2 PMC7973794 33737682

[16] Nakamoto S, Shien T, Iwamoto T, Kubo S, Yamamoto M, Yamashita T, et al. Absolute lymphocyte count and neutrophil-to-lymphocyte ratio as predictors of CDK 4/6 inhibitor efficacy in advanced breast cancer. Sci Rep. 2024;14:9869. 10.1038/s41598-024-60101-x PMC11059159 38684839

[17] Mitsuyoshi A, Nagahashi M, Kanaoka H, Oshiro A, Togashi Y, Hattori A, et al. Neutrophil-to-lymphocyte ratio at the end of treatment with CDK4/6 inhibitors is an independent prognostic factor for ER-positive HER2-negative advanced breast cancer. Int J Clin Oncol. 2024;29:1850–9. 10.1007/s10147-024-02625-w 39278979

[18] Allison KH, Hammond MEH, Dowsett M, McKernin SE, Carey LA, Fitzgibbons PL, et al. Estrogen and progesterone receptor testing in breast cancer: ASCO/CAP guideline update. J Clin Oncol. 2020;38:1346–66. 10.1200/JCO.19.02309 31928404

[19] Wolff AC, Hammond MEH, Allison KH, Harvey BE, Mangu PB, Bartlett JMS, et al. Human epidermal growth factor receptor 2 testing in breast cancer: American Society of Clinical Oncology/College of American Pathologists clinical practice guideline focused update. J Clin Oncol. 2018;36:2105–22. 10.1200/JCO.2018.77.8738 29846122

[20] Togashi Y, Nagahashi M, Oshiro A, Sugimoto G, Mitsuyoshi A, Kanaoka H, et al. Clinical implications of peripheral blood biomarkers in patients with advanced breast cancer treated with trastuzumab emtansine and trastuzumab deruxtecan. Int J Clin Oncol. 2025;30:1331–40. 10.1007/s10147-025-02768-4 PMC12187903 40295416

[21] Valle I, Grinda T, Antonuzzo L, Pistilli B. Antibody-drug conjugates in breast cancer: mechanisms of resistance and future therapeutic perspectives. npj Breast Cancer. 2025;11:102. 10.1038/s41523-025-00829-5 PMC12480482 41022825

[22] Koh CH, Bhoo-Pathy N, Ng KL, Jabir RS, Tan GH, See MH, et al. Utility of pre-treatment neutrophil-lymphocyte ratio and platelet-lymphocyte ratio as prognostic factors in breast cancer. Br J Cancer. 2015;113:150–8. 10.1038/bjc.2015.183 PMC4647546 26022929

[23] Zhang M, Huang XZ, Song YX, Gao P, Sun JX, Wang ZN. High platelet-to-lymphocyte ratio predicts poor prognosis and clinicopathological characteristics in patients with breast cancer: A meta-analysis. BioMed Res Int. 2017;2017:9503025. 10.1155/2017/9503025 PMC5610825 29082257

[24] Savioli F, Morrow ES, Dolan RD, Romics L, Lannigan A, Edwards J, et al. Prognostic role of preoperative circulating systemic inflammatory response markers in primary breast cancer: meta-analysis. Br J Surg. 2022;109:1206–15. 10.1093/bjs/znac319 36130112

[25] Obeagu EI. Inflammatory blood markers in breast cancer: a narrative review from early detection to therapy response. Ann Med Surg (Lond). 2025;87:5906–11. 10.1097/MS9.0000000000003687 PMC12401255 40901118

[26] Nicolò E, Giugliano F, Ascione L, Tarantino P, Corti C, Tolaney SM, et al. Combining antibody-drug conjugates with immunotherapy in solid tumors: current landscape and future perspectives. Cancer Treat Rev. 2022;106:102395. 10.1016/j.ctrv.2022.102395 35468539

[27] Nucera S, Conti C, Martorana F, Wilson B, Genta S. Antibody-drug conjugates to promote immune surveillance: lessons learned from breast cancer. Biomedicines. 2024;12:1491. 10.3390/biomedicines12071491 PMC11274676 39062065

[28] Ogitani Y, Aida T, Hagihara K, Yamaguchi J, Ishii C, Harada N, et al. DS-8201a, A novel HER2-targeting ADC with a novel DNA topoisomerase I inhibitor, demonstrates a promising antitumor efficacy with differentiation from T-DM1. Clin Cancer Res. 2016;22:5097–108. 10.1158/1078-0432.CCR-15-2822 27026201

[29] Kepp O, Kroemer G. Immunogenic cell death and bystander killing: expanding the therapeutic potential of modern antibody-drug conjugates. Oncoimmunology. 2025;14:2533488. 10.1080/2162402X.2025.2533488 PMC12269677 40662767

[30] Shi R, Jia L, Lv Z, Cui J. Another power of antibody-drug conjugates: immunomodulatory effect and clinical applications. Front Immunol. 2025;16:1632705. 10.3389/fimmu.2025.1632705 PMC12404974 40909270

[31] Ogitani Y, Hagihara K, Oitate M, Naito H, Agatsuma T. Bystander killing effect of DS-8201a, a novel anti-human epidermal growth factor receptor 2 antibody-drug conjugate, in tumors with human epidermal growth factor receptor 2 heterogeneity. Cancer Sci. 2016;107:1039–46. 10.1111/cas.12966 PMC4946713 27166974

[32] Giugliano F, Corti C, Tarantino P, Michelini F, Curigliano G. Bystander effect of antibody-drug conjugates: Fact or fiction? Curr Oncol Rep. 2022;24:809–17. 10.1007/s11912-022-01266-4 35305211

[33] Imamura M, Morimoto T, Egawa C, Fukui R, Bun A, Ozawa H, et al. Significance of baseline neutrophil-to-lymphocyte ratio for progression-free survival of patients with HER2-positive breast cancer treated with trastuzumab emtansine. Sci Rep. 2019;9:1811. 10.1038/s41598-018-37633-0 PMC6372578 30755651

