# Supplementary material for: Baseline peripheral blood immune markers associated with clinical outcomes in patients with advanced breast cancer treated with trastuzumab deruxtecan
Source: BMC Cancer. 2026 Jul 13;26:1201. doi: 10.1186/s12885-026-16548-4 (PMC13644231; doi:10.1186/s12885-026-16548-4)
Supplement: Supplementary file 1 [file 12885_2026_16548_MOESM1_ESM.docx]

**Additional Material**

**Baseline peripheral blood immune markers associated with clinical outcomes in patients with advanced breast cancer treated with trastuzumab deruxtecan**

**Supplementary Tables**

**Supplementary Table S1. Reasons for discontinuation of trastuzumab deruxtecan according to baseline absolute lymphocyte count and neutrophil-to-lymphocyte ratio status**

| Reason for discontinuation | Overall  (*n*=98) | Low ALC  (*n*=68) | High ALC  (*n*=30) | Low NLR  (*n*=53) | High NLR  (*n*=45) |
| --- | --- | --- | --- | --- | --- |
| Disease progression  Adverse events  Death  Others | 71 (72.4%)  17 (17.3%)  1 (1.0%)  9 (9.2%) | 49 (72.1%)  10 (14.7%)  1 (1.5%)  8 (11.8%) | 22 (73.3%)  7 (23.3%)  0 (0.0%)  1 (3.3%) | 38 (71.7%)  13 (24.5%)  0 (0.0%)  2 (3.8%) | 33 (73.3%)  4 (8.9%)  1 (2.2%)  7 (15.6%) |

Abbreviations: ALC, absolute lymphocyte count; NLR, neutrophil-to-lymphocyte ratio.

**Supplementary Table S2. Univariate and multivariate analyses of baseline neutrophil-to-lymphocyte ratio and time-to-treatment failure**

| Variables | Univariate | | | Multivariate | | |
| --- | --- | --- | --- | --- | --- | --- |
|  | HR | 95% CI | *P*-value | HR | 95% CI | *P*-value |
| **Time-to-treatment failure**  Age (<50 vs. ≥50 years)  ECOG PS (0–1 vs. others)  Diagnosis (Recurrence vs. *De novo*)  ER status (+/-)  HER2 status (HER2-low vs. HER2-positive)  Number of prior treatment regimens for metastatic disease (≥3 vs. 0–2)  Prior perioperative chemotherapy (Yes vs. No)  Prior trastuzumab/pertuzumab exposure (Yes vs. No)  Prior T-DM1 exposure (Yes vs. No)  Prior CDK4/6 inhibitor exposure (Yes vs. No)  Endocrine resistance status (Yes vs. No)  Liver metastases (+/-)  Lung or pleural metastases (+/-)  Brain metastases (+/-)  ALC (Low vs. High)  NLR (High vs. Low)  PLR (High vs. Low) | 0.99  0.59  0.83  1.31  2.27  1.09  0.96  0.41  0.63  1.70  1.67  1.07  1.02  1.01  1.77  1.53  1.68 | 0.60–1.65  0.35–0.98  0.56–1.24  0.82–2.11  1.48–3.48  0.73–1.63  0.64–1.43  0.27–0.64  0.40–0.98  1.10–2.64  1.10–2.55  0.72–1.59  0.68–1.52  0.59–1.73  1.14–2.75  1.02–2.30  1.09–2.58 | 0.9821  **0.0432**  0.3673  0.2566  **0.0002**  0.6836  0.8301  **<0.0001**  **0.0421**  **0.0177**  **0.0162**  0.7546  0.9206  0.9752  **0.0105**  **0.0381**  **0.0185** | 0.98  0.73  1.12  1.25  1.23  0.41  0.66  1.06  1.63 | 0.58–1.66  0.43–1.25  0.72–2.00  0.46–3.39  0.73–2.08  0.15–1.11  0.33–1.29  0.52–2.14  1.06–2.49 | 0.9416  0.2579  0.4943  0.6550  0.4379  0*.0794*  0.2204  0.8786  **0.0257** |

Abbreviations: ALC, absolute lymphocyte count; CDK4/6, cyclin-dependent kinase 4 and 6; CI, confidence interval; ECOG PS, Eastern Cooperative Oncology Group performance status; ER, estrogen receptor; HER2, human epidermal growth factor receptor 2; HR, hazard ratio; NLR, neutrophil-to-lymphocyte ratio; OS, overall survival; PLR, platelet-to-lymphocyte ratio; T-DM1, trastuzumab emtansine.

**Supplementary Table S3. Univariate and multivariate analyses of baseline neutrophil-to-lymphocyte ratio and overall survival**

| Variables | Univariate | | | Multivariate | | |
| --- | --- | --- | --- | --- | --- | --- |
|  | HR | 95% CI | *P*-value | HR | 95% CI | *P*-value |
| **Overall survival**  Age (<50 vs. ≥50 years)  ECOG PS (0–1 vs. others)  Diagnosis (Recurrence vs. *De novo*)  ER status (+/-)  HER2 status (HER2-low vs. HER2-positive)  Number of prior treatment regimens for metastatic disease (≥3 vs. 0–2)  Prior perioperative chemotherapy (Yes vs. No)  Prior trastuzumab/pertuzumab exposure (Yes vs. No)  Prior T-DM1 exposure (Yes vs. No)  Prior CDK4/6 inhibitor exposure (Yes vs. No)  Endocrine resistance status (Yes vs. No)  Liver metastases (+/-)  Lung or pleural metastases (+/-)  Brain metastases (+/-)  ALC (Low vs. High)  NLR (High vs. Low)  PLR (High vs. Low) | 1.88  0.51  0.75  1.05  2.08  1.05  0.78  0.46  0.88  1.36  1.29  2.34  1.11  1.57  2.68  2.16  1.41 | 0.96–3.66  0.21–1.25  0.40–1.42  0.53–2.10  0.97–4.42  0.55–1.98  0.41–1.49  0.22–0.97  0.42–1.85  0.63–2.96  0.65–2.55  1.21–4.50  0.59–2.12  0.74–3.32  1.28–5.61  1.14–4.11  0.73–2.73 | *0.0649*  0.1412  0.3765  0.8861  *0.0582*  0.8922  0.4463  **0.0407**  0.7289  0.4345  0.4703  **0.0112**  0.7405  0.2396  **0.0089**  **0.0180**  0.3108 | 1.58  0.61  1.17  1.11  1.00  0.22  0.39  1.34  1.90  2.02 | 0.75–3.34  0.24–1.55  0.56–2.45  0.18–6.86  0.45–2.26  0.04–1.13  0.10–1.52  0.41–4.32  0.90–4.02  1.02–3.96 | 0.2310  0.2999  0.6843  0.9073  0.9821  *0.0706*  0.1744  0.6268  *0.0911*  **0.0423** |

Abbreviations: ALC, absolute lymphocyte count; CDK4/6, cyclin-dependent kinase 4 and 6; CI, confidence interval; ECOG PS, Eastern Cooperative Oncology Group performance status; ER, estrogen receptor; HER2, human epidermal growth factor receptor 2; HR, hazard ratio; NLR, neutrophil-to-lymphocyte ratio; OS, overall survival; PLR, platelet-to-lymphocyte ratio; T-DM1, trastuzumab emtansine.

**Supplementary Table S4. Sensitivity analyses of baseline peripheral blood immune markers modeled as continuous variables for clinical outcomes**

| Variables | HR | 95% CI | *P*-value |
| --- | --- | --- | --- |
| **Time-to-treatment failure**  ALC (per 100 cells/μL increase)  NLR (per 1-unit increase)  PLR (per 10-unit increase) | 0.95  1.08  1.02 | 0.91–0.98  1.02–1.12  1.00–1.03 | **0.0052**  **0.0042**  **0.0287** |
| **Overall survival**  ALC (per 100 cells/μL increase)  NLR (per 1-unit increase)  PLR (per 10-unit increase) | 0.92  1.10  1.03 | 0.87–0.98  1.01–1.16  1.00–1.04 | **0.0111**  **0.0079**  **0.0147** |

Abbreviations: ALC, absolute lymphocyte count; NLR, neutrophil-to-lymphocyte ratio; PLR, platelet-to-lymphocyte ratio. Hazard ratios are presented per 100 cells/μL increase for ALC, per 1-unit increase for NLR, and per 10-unit increase for PLR.

**Supplementary Figures**

**Supplementary Figure S1. Association between baseline peripheral blood immune markers and clinical outcomes in patients treated with T-DXd in HER2-positive disease subgroup.**
(A–C) Kaplan–Meier curves of time-to-treatment failure (TTF) according to baseline absolute lymphocyte count (ALC; high vs. low), neutrophil-to-lymphocyte ratio (NLR; high vs. low), and platelet-to-lymphocyte ratio (PLR; high vs. low). High and low groups were defined by the pre-specified cutoff values, resulting in the following numbers of patients in each group: ALC high, n=27; ALC low, n=40; NLR high, n=29; NLR low, n=38; PLR high, n=35; PLR low, n=32. (D–F) Kaplan–Meier curves of overall survival (OS) stratified by the same ALC, NLR, and PLR categories used in panels A–C. *P* values were calculated using the log-rank test. T-DXd, trastuzumab deruxtecan; HER2, human epidermal growth factor receptor 2.

**Supplementary Figure S2. Association between baseline peripheral blood immune markers and clinical outcomes in patients treated with T-DXd in HER2-low ER-positive disease subgroup.**
(A–C) Kaplan–Meier curves of time-to-treatment failure (TTF) according to baseline absolute lymphocyte count (ALC; high vs. low), neutrophil-to-lymphocyte ratio (NLR; high vs. low), and platelet-to-lymphocyte ratio (PLR; high vs. low). High and low groups were defined by the pre-specified cutoff values, resulting in the following numbers of patients in each group: ALC high, n=9; ALC low, n=38; NLR high, n=18; NLR low, n=29; PLR high, n=33; PLR low, n=14. (D–F) Kaplan–Meier curves of overall survival (OS) stratified by the same ALC, NLR, and PLR categories used in panels A–C. *P* values were calculated using the log-rank test. T-DXd, trastuzumab deruxtecan; HER2, human epidermal growth factor receptor 2; ER, estrogen receptor.

**Supplementary Figure S3. Association between baseline peripheral blood immune markers and clinical outcomes in patients treated with T-DXd in HER2-low triple-negative disease subgroup.**
(A–C) Kaplan–Meier curves of time-to-treatment failure (TTF) according to baseline absolute lymphocyte count (ALC; high vs. low), neutrophil-to-lymphocyte ratio (NLR; high vs. low), and platelet-to-lymphocyte ratio (PLR; high vs. low). High and low groups were defined by the pre-specified cutoff values, resulting in the following numbers of patients in each group: ALC high, n=4; ALC low, n=8; NLR high, n=9; NLR low, n=3; PLR high, n=11; PLR low, n=1. (D–F) Kaplan–Meier curves of overall survival (OS) stratified by the same ALC, NLR, and PLR categories used in panels A–C. *P* values were calculated using the log-rank test. T-DXd, trastuzumab deruxtecan; HER2, human epidermal growth factor receptor 2.
